# Recognizing and Resisting Ageism: Views from Older Adults

**DOI:** 10.1093/geroni/igaf122.3647

**Published:** 2025-12-31

**Authors:** Aaron Li, Natalie Galucia, Michele Dinman, Brian Carpenter, Nancy Morrow-Howell

**Affiliations:** Washington University, St. Louis, Missouri, United States; Washington University, Saint Louis, Missouri, United States; Washington University, St. Louis, Missouri, United States; Washington University, St. Louis, Missouri, United States; Washington University, St. Louis, Missouri, United States

## Abstract

This study aims to generate knowledge for the development of anti-ageism interventions for older people. We conducted 12 focus groups (N = 75) to explore experiences with interpersonal and internalized ageism, reactions to ageist incidents, and ideas about resisting. We conducted six-month follow-up survey interviews to gain understanding about how focus group discussions changed awareness and actions regarding ageism. Participants ranged from 60 to 91 years (M = 75); 76% were female, 56% White, 41% Black, and 20% identified as LGBTQIA+. Thematic analysis revealed that not all participants understand the concept of ageism, especially internalized ageism. Among those who did, lack of respect and feeling invisible were identified as hallmarks. Ageism most often appeared among families and in businesses and took the form of disregard and paternalism. Fewer examples were provided in health care and employment, although harmful incidents mentioned. Internalized ageism wasn’t well understood; participants often talked about the challenges of aging instead. For participants with minoritized identities, racism, ableism, and homophobia played a more central role than ageism in experiences of discrimination. Reactions to ageism ranged from nonresponse to context-dependent actions shaped by relationships with perpetrators. In ongoing survey follow-ups (n = 52 to date), 75% shared new examples of ageism; 63% described new examples of internalized ageism; 51% reported a conversation about ageism with someone; 38% had addressed an ageist comment directed toward them; and 84% reported increased awareness. These findings suggest that simple discussions about ageism can serve as an intervention, raising awareness and encouraging resistance.

